# Characterization of a Ferret Model of Traumatic Brain Injury due to Under‐Vehicle Blast, Controlled Cortical Impact, or Blast Plus Impact

**DOI:** 10.1002/jnr.70081

**Published:** 2025-09-15

**Authors:** Molly J. Goodfellow, Amanda L. Hrdlick, Boris Piskoun, Julie L. Proctor, Parisa Rangghran, Michael C. Shaughness, Alexandra Vesselinov, Su Xu, Rao P. Gullapalli, Ulrich H. Leiste, William L. Fourney, Catriona H. T. Miller, Jody C. Cantu, Gary Fiskum

**Affiliations:** ^1^ Department of Anesthesiology and the Center for Shock, Trauma and Anesthesiology Research (STAR) University of Maryland School of Medicine Baltimore Maryland USA; ^2^ Department of Diagnostic Radiology University of Maryland School of Medicine Baltimore Maryland USA; ^3^ Department of Aerospace Engineering University of Maryland School of Engineering College Park Maryland USA; ^4^ Soldier Treatment & Evacuation, Soldier Medical Devices, PEO Soldier, US Army Medical Materiel Development Activity Fort Detrick Maryland USA; ^5^ Air Force Research Laboratory, 711th Human Performance Wing, Air and Space Biosciences Division, En Route Care Section, US air Force Materiel Command Baltimore Maryland USA

**Keywords:** axonal injury, blood–brain barrier, gyrencephalic, inflammation

## Abstract

Under‐vehicle blast (UVB) generated from landmines is a unique traumatic brain injury (TBI) mechanism affecting warfighters. UVB hyperacceleration can result in injury independent of impact; however, a secondary impact injury can also occur. To date, translation of findings from rodent TBI models to improved patient outcomes has been unsuccessful, perhaps due to neuroanatomical differences between humans and rodents, including white‐to‐gray matter ratio and cortical gyrification. To address this modeling difference, a UVB model was developed in ferrets, the brains of which more closely resemble humans. Male ferrets underwent UVB‐alone (Blast), controlled cortical impact (CCI)‐alone, combined UVB + CCI (BCCI), or craniotomy (Sham) procedures. Neurobehavioral assays were optimized and used to assess mood, memory, and motor control. Blast and BCCI ferrets underwent neuroimaging at baseline and 7 days post‐injury. All ferrets were euthanized by terminal perfusion with paraformaldehyde on day 7 for histologic analysis. Results indicate that UVB alters cortical metabolites and induces blood–brain barrier (BBB) disruption. CCI leads to BBB disruption and cortical diffuse axonal injury, but this is not exacerbated by combination with UVB. BCCI does result in several alterations in key cortical metabolites indicative of increased neuronal injury, oxidative stress, and glial activation as well as impaired neurotransmission and energy generation. Additionally, BCCI significantly increases hyperactivity and impairs spatial memory. Anxiety‐like behavior, mood, and motor function approached statistical significance. Taken together, we provide a military‐relevant model of UVB in a gyrencephalic animal, the ferret, that may be applied in future investigations into TBI pathophysiology and potential treatment.

## Graphical abstract

Graphical abstract depicting the outcomes measured in a ferret model of under‐vehicle blast + impact injury: imaging, histopathology, and neurobehavior. Created in https://BioRender.com.

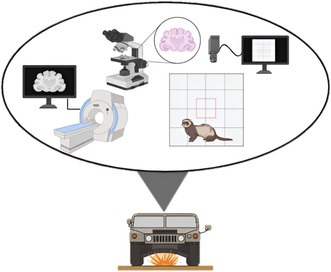

**Summary**

- Ferrets, which possess brains that more closely resemble the human brain than animals typically used for research (i.e., rodents), are an emerging model species for neuroscience research.
- This paper provides methodology for investigating structural and functional changes that occur after different types of traumatic brain injury in the ferret, including behavioral assessment, neuroimaging, and postmortem analyses.
- The methods described may be applied to future research using ferrets including studies of neurologic disease pathology as well as testing the efficacy of potential therapeutics.

## 1 Introduction

Twenty percent of military personnel serving in recent conflicts have suffered some form of traumatic brain injury (TBI). Approximately 80% of mild TBI incurred in the combat theater is linked to blast exposure (Rigg and Mooney 2011). Neuroimaging studies in blast TBI patients have shown blast can induce intracranial hemorrhage, cerebral edema, and changes in white matter, including diffuse axonal injury (Magnuson et al. 2012; Davenport et al. 2012; Saar‐Ashkenazy et al. 2023). In addition to gross neuropathology, blast exposure is associated with long‐term neurobehavioral changes, including post‐traumatic stress disorder, cognitive problems, and substance abuse (Saar‐Ashkenazy et al. 2023; Goldstein et al. 2012; Hoge et al. 2008; Belding et al. 2021). However, most clinical research simply refers to “blast” and does not distinguish between blast types. Animal models have focused primarily on “blast overpressure,” which is delivered to an immobilized subject in a shock tube. It should be noted, however, that the forces exerted on the brain during under‐vehicle blast (UVB) also include hyperacceleration. Previous work from our lab investigated a rodent model of under‐vehicle blast‐induced TBI (Proctor et al. 2014, 2017; Tchantchou et al. 2017, 2018) where we noted that UVB‐induced hyperacceleration led to axonal injury and neurobehavioral impairments.

Rodents have been used for decades to study the pathophysiological mechanisms and neurobehavioral deficits following TBI and develop mitigation strategies. However, rodents are lissencephalic with relatively little white matter, whereas humans are gyrencephalic with a large white‐to‐gray matter ratio. Given that white matter is particularly sensitive to blast injury and the presence of cortical gyrification influences mechanical stress points, the translation of findings from rodents into clinical practice is challenging (Vink 2018). Large animal species may be superior models for brain injury research, given their gyrencephaly and extensive white matter. However, unlike swine, sheep, and primates, ferrets grow to a weight of ≤ 2 kg and are amenable to a variety of behavioral and magnetic imaging assessments, thereby facilitating access to chronic studies with a variety of outcome measures (Hutchinson et al. 2018; Obasa et al. 2022; Schwerin et al. 2023, 2021; Goodfellow et al. 2022).

In this pilot study, we developed a gyrencephalic ferret model of under‐vehicle blast TBI. We first characterized models of under‐vehicle blast and cortical impact individually before combining the two, simulating a compound injury that results from exposure to improvised explosive device (IED) detonation and subsequent head impact with the vehicle roof or window. We hypothesized that blast‐alone, impact‐alone, and blast + impact will all result in unique neurologic outcomes at a subacute timepoint of 7 days post‐injury.

## 2 Methods

The study protocol was reviewed and approved by the University of Maryland, Baltimore Institutional Animal Care and Use Committee (0620009) and the Air Force Medical Readiness Agency (FWR‐2020‐0015A). Animals were handled, and studies were conducted, under a program of animal care accredited by the Association for Assessment and Accreditation of Laboratory Animal Care International and in accordance with the National Research Council's 2011 Guide for the Care and Use of Laboratory Animals (in compliance with Department of Defense Instruction 3216.1). Thirty‐three male sable ferrets (~10 weeks of age; Marshall Farms, North Rose, NY) were assigned to one of four groups: under‐vehicle blast‐alone (Blast; *n* = 12), controlled cortical impact‐alone (CCI; *n* = 8), blast + CCI (BCCI; *n* = 8), or Sham (*n* = 5). As this was a pilot study to develop the model, no power analyses were performed to determine group sizes. Instead, sample sizes for this pilot study were estimated based on previous experience with the models and were chosen to represent no less than 10% of the projected sample size of the main data set. Behavioral assessments were completed 6 days post‐injury. Imaging was performed up to 1 week prior to injury and again 1 week post‐injury. Animals were pair‐housed until injury, then individually housed until euthanasia at 7 days post‐injury to prevent wound dehiscence by rough and tumble play. Animal care staff provided standardized enrichment including ferret treats, chew toys, bedding/digging media, and hammocks or igloos.

### 2.1 Under‐Vehicle Blast

**FIGURE 1:**
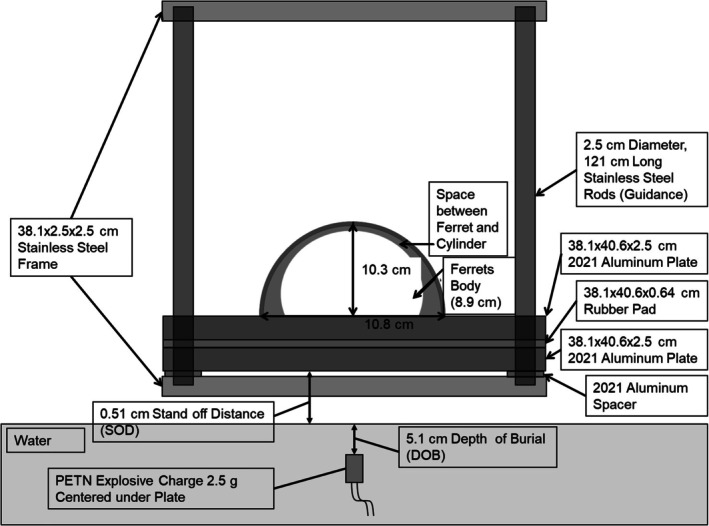
Test set‐up with specific dimensions for testing ferrets using an explosive charge for acceleration. Animals were loaded into the 10.3 cm space between the cylinder and the top of the superior aluminum plate. The explosive was centered to the plates, 5.1 cm under the water surface (0.51 cm stand off distance between the bottom of the inferior aluminum plate and the water surface). The explosive was detonated and the restrained ferret, aluminum plates, and rubber pad were accelerated vertically, guided up and down by the stainless steel rods.

Blast and BCCI ferrets (~1000 g) were sedated 15–30 min prior to blast with an intraperitoneal injection of 0.05 mg/kg of dexmedetomidine (Dexdomitor, Zoetis), then anesthetized with 4% isoflurane in 45% O_2_ in medical air in an induction chamber for 4–5 min. Anesthetized ferrets were loaded into an individual custom‐made polycarbonate cylinder restrainer (12.7 × 40.6 cm) in a prone position affixed to two top 2.5 cm thick aluminum blast plates (40.6 × 38 cm) separated by a 6 cm thick rubber pad of the same dimensions. Pentaerythritol tetranitrate (PETN; 1.75–2.5 g) was submerged in water at a depth of 2 in. An air gap was left between the water surface and the bottom plates (0.2 in) to avoid transfer of the shockwave directly into the plate. PETN was provided and detonated by a licensed explosive expert under the center of the blast plates suspended above a filled polyurea coated steel water tank (1.2 × 0.6 × 1.2 m), causing the plates, pad, and cylinder containing the animal to travel vertically to heights of approximately 1 m guided by poles located in holes in each corner of the plates and pad (Proctor et al. 2014, 2017). Peak acceleration (*g*) and jerk (m/s^3^) were recorded for each blast. See Figure 1. Immediately following detonation, ferrets were removed from the restrainers and BCCI animals proceeded to CCI surgery.

### 2.2 Controlled Cortical Impact

Ferrets were sedated and anesthetized as above and were secured within a stereotactic frame with ear bars (Kopf, Tujunga, CA) where they were maintained on a 1.5%–2% isoflurane and 45% O_2_ in medical air via a nose cone. Rectal temperature was kept at 37°C± 0.5°C with a homeothermic blanket system (Harvard Apparatus, Holliston, MA) and heart rate, respiratory rate, and blood oxygen saturation (SpO_2_) were monitored (Biopac Systems Inc) for the duration of anesthesia, approximately one hr. Following aseptic preparation, a midline dorsal incision was made 3–4 cm over the calvarium to visually identify the junction of the supraorbital crest as a landmark on the skull. A surgical marking pen was then used to mark the center of the craniotomy location at a position 15 mm caudal and 6 mm lateral to the landmark. A 6–7 mm wide craniotomy was established with a surgical bone microdrill (Foredom) between the coronal, lambdoid, and sagittal sutures, followed by induction of moderate brain injury using a CCI device (Impact One, Leica Biosystems) with a 5 mm beveled piston (Goodfellow et al. 2022; Schwerin et al. 2017). An impact resulting in dura rupture was delivered at a 6.0 mm depth with 6.0 m/s velocity and 50 ms duration. Surgifoam (Ethicon) was placed over the craniotomy as a skull cap replacement and secured with dental cement and the incision sutured shut. Sham animals underwent procedures identical to BCCI (including sedation/anesthesia, placement in the blast restrainer, and craniotomy surgery), except no explosive was detonated and no impact was made.

Atipamezole (0.05 mg/kg) was injected intraperitoneally immediately after the completion of blast (blast‐alone) or surgical (CCI, BCCI, Sham) procedures to reverse dexmedetomidine sedation. Buprenorphine (0.01–0.05 mg/kg) was administered subcutaneously to all animals for analgesia every 8–12 h for 3 days, with the first dose administered prior to surgical manipulation. Animals were monitored twice daily for the first 3 days, then daily until their experimental timepoint was reached.

### 2.3 Neurobehavioral Testing

All tests were performed in a dedicated ferret behavior suite (3.9 × 7.1 m). Prior to any experimental manipulation or baseline testing, animals were habituated to the room twice by allowing them to explore for 30 min with their cage mate. During this exploration and the observation period described below, animals had access to a standard set of enrichment items, including boxes, tubes, and balls. Enrichment items, testing objects, and apparatuses were cleaned between animals with 70% ethanol.

#### 2.3.1 Observation of Play Behavior

No more than 1 week before injury and on 6 days post‐injury, animals were transported individually to the behavior suite in a covered pet carrier. The carrier was placed on the ground and the door opened. Animal behavior was filmed for 5 min (GoPro Hero Black 8); videos were analyzed with Behavioral Observation Research Interactive Software (BORIS) (Friard and Gamba 2016) for latency to exit the carrier and to express play behaviors (see ethogram in Table 1). An increased latency to exit and/or express these behaviors may be associated with changes in psychological well‐being, such as increases in depressive‐like and/or anxiety‐like behavior (Vinke and Schoemaker 2012; Oliveira et al. 2010; Pellis et al. 2015; Jain et al. 2012). After the filming period, animals were allowed to exercise for an additional 25 min before continuing with other behavior tests.

**TABLE 1:** Behavioral observation ethogram.

Behavior	Description
Dooking	The animal emits a series of hoot/chuckle vocalizations. Excludes single or very short (< 2 s) strings of vocalizations
Postural change	The animal changes from a flat back to an arched back
Piloerection	The animal's fur stands on end on its tail and/or back
Weasel war dance	The animal starts bouncing from side to side. They may shake their head or fall over. Excludes running or galloping
Open mouth	The animal opens its mouth. The upper teeth must be covered by the top lip

#### 2.3.2 Open Field (OF)

On 6 days post‐injury, animals were placed into the Plexiglas arena (122 × 122 × 77 cm) and allowed to explore for 10 min. AnyMaze (Ethovision) was used to record and analyze behavior for distance traveled and time spent in different zones of the arena.

#### 2.3.3 Object Location Test and Novel Object Recognition (OLT/NOR)

Fifteen minutes after OF, animals proceeded through the three‐phase OLT/NOR (Denninger et al. 2018) in the OF arena. Each phase lasted 10 min and was separated in time by a 1‐h period of rest in the pet carrier where animals had *ad libitum* access to food and water. For the training phase, animals were allowed to explore two identical cuboid aluminum weighted tins (approximately 1 L volume) placed opposite each other 30 cm from the wall of the arena. During the OLT phase, one of these tins (the “moved object”) was moved to the opposite side of the arena. During the NOR phase, the unmoved object was replaced with a round 1 L plastic bottle filled with water mixed with nontoxic yellow paint and sealed with a blue cap. AnyMaze was used for recording and BORIS was used for analysis of time spent with each object. The percentage of time spent with the moved or novel object relative to total object exploration time (preference index; PI) was calculated from BORIS observations.

#### 2.3.4 Ladder Walk

Construction of the horizontal ladder apparatus and procedures was based on Metz and Whishaw (2002). The apparatus consisted of clear Plexiglass walls (2.4 m long) in which a row of holes was drilled 5 cm from the bottom and 1.5 cm apart. Round carbon fiber rods (0.125 cm in diameter) were passed through the holes to connect the walls. An acrylic roof spanned the length of the apparatus; the width and height of the alley were adjusted to animal size so that animals could walk comfortably with a straight back but to discourage galloping. A reward box containing toys was placed at the end of the apparatus as an incentive to cross. All four limbs were filmed simultaneously with a GoPro positioned at a slight ventral angle to the ladder.

No more than 1 week before injury, animals were trained to walk across the ladder. Performance was determined from a minimum of 100 steps in a separate pre‐injury baseline session as well as on 6 days post‐injury. Recordings were analyzed frame by frame at 30 frames/s with BORIS. A slip score was calculated from the number and severity of slips per step as described by Metz and Whishaw (2002).

### 2.4 Neuroimaging

Scans were performed in a subset of animals (*n* = 5 Blast and *n* = 5 BCCI) at baseline (no more than 1 week before injury) and at 7 days post‐injury. Prior to imaging, animals were sedated with dexmedetomidine and anesthetized with isoflurane as above.

MR studies were performed on a 7‐Tesla, 30‐cm bore Bruker AVANCE system (Bruker Biospin MRI, Ettlingen, Germany), which interfaced to a Bruker Paravision 6.0 console and was equipped with a Bruker 86‐mm circular‐polarized RF volume coil that worked as a transmitter and a Bruker 30‐mm surface coil as a receiver. Image acquisition parameters and analysis methods were similar to those reported in a prior study (Goodfellow et al. 2022). Briefly, coronal T_2_‐weighted Bruker rapid imaging with refocused echoes (RARE) images were obtained (spin‐echo, repetition time (TR)/echo time (TE) 3200/33 ms, 1 average, RARE factor 4, field of view (FOV) 55 × 55 mm^2^, in‐plane resolution 0.15 × 0.15 mm^2^, number of slices 18, slice thickness 1.5 mm, with an acquisition time 5 min). These images served to determine abnormalities in T_2_‐weighted signal intensity.

In vivo proton spectra were obtained from the left cortex (5 × 2.5 × 5 mm^3^) using a proton short‐TE Point‐RESolved Spectroscopy (PRESS) pulse sequence (Xu et al. 2013), TR/TE = 2500/10 ms, and 400 averages. The unsuppressed water signal from the prescribed voxels was obtained to serve as a reference for determining metabolite concentrations. Quantification of the MRS was based on frequency domain analysis using a “Linear Combination of Model spectra” (LCModel) (Provencher 1993). Absolute concentrations were estimated with the LCModel automatic procedure (version 6.3‐0G). Only metabolites that passed the Cramer‐Rao lower bound of ≤ 35% were considered for analysis.

### 2.5 ImageJ Analyses of T_2_ ‐Weighted Hyperintensities

Edematous hyperintensity volumes within the cortex were identified from a series of 1 mm thick T_2_‐weighted images. Cortical areas on T_2_ images appearing bright white were classified as “hyperintensities” (HI) and areas appearing matte gray were classified as “healthy”. Areas quantified from each section were summed to calculate cortical volumes (mm^3^) at each of the time points using ImageJ software (Schneider et al. 2012).

### 2.6 Euthanasia

At 7 days post‐injury, ferrets were deeply anesthetized with a ketamine/xylazine/acepromazine cocktail and euthanized via transcardial perfusion of oxygenated artificial cerebrospinal fluid (148 mM NaCl, 5.0 mM glucose, 3.0 mM KCl, 1.85 mM CaCl_2_, 1.7 mM MgCl_2_, 1.5 mM Na_2_HPO_4_, and 0.14 mM NaH_2_PO_4_ [pH 7.4]) followed by 4% paraformaldehyde, then 1% paraformaldehyde/5% sucrose solution. Brains were removed from the skull and postfixed in 4% paraformaldehyde for 48 h, followed by fixation in 30% sucrose prior to sectioning. A freezing microtome was used to slice coronal sections (40 μm). Slices were stored in a cryoprotectant antifreeze solution consisting of glycerol, ethylene glycol, distilled water, and phosphate buffered saline (3:3:3:1 volume ratio) at −20°C until further processing.

### 2.7 Immunohistochemistry

Free‐floating sections were incubated in 1% sodium borohydride for 20 min, followed by rinsing with Tris‐buffered saline (TBS). Then, tissue was incubated in primary antibody in TBS + 0.4% triton X‐100 at room temperature overnight for the evaluation of inflammation (rabbit anti‐Iba1,1:20,000, Wako), blood‐brain barrier dysfunction (goat anti‐immunoglobulin G (IgG), 1:300,000, Novus), and diffuse axonal injury (rabbit anti‐amyloid precursor protein (APP),1:10,000, Invitrogen). Sections were rinsed with TBS before and after a 1 h incubation with corresponding biotinylated anti‐rabbit and anti‐mouse secondary antibody (1:600, Vector Laboratories, Burlingame, CA), followed by 1 h in Vectastain ABC kit (Vector Laboratories, Burlingame, CA). Sections were washed with 0.175 M sodium acetate, followed by 15 min incubation in nickel diaminobenzidine solution. The reaction was terminated by rinsing with 0.175 M sodium acetate and then TBS. Finally, sections were mounted onto gelatin‐coated slides and allowed to dry overnight. Slides were dehydrated in ethanol and xylene and cover slipped with DPX mounting media.

### 2.8 Stereological Quantification

Separate sets of tissue were immunolabeled for neuroinflammation (Iba1), blood brain barrier disruption (IgG) and diffuse axonal injury (APP). We examined 5–7 sections, spaced 960 μm apart per marker. These sections were selected to cover the primary injury site in the cortex, as well as three sections anterior and three posterior to it. Quantitative analysis was conducted using the Cavalieri method and a Nikon E800 microscope at 10× magnification. StereoInvestigator software (MBF Biosciences, VT) was used to randomly generate and superimpose a 25 μm × 25 μm grid over the cortical region of interest. Areas of IgG+, Iba1+, or APP+ immunoreactivity (defined as being covered by black reactive product from diaminobenzidine) were manually marked on the grid by a treatment‐blinded investigator. Volumes (mm^3^) were calculated using the software (Volume = Total number of grid points marked × Distance between points in XY × Distance between points in Z) (Altunkaynak et al. 2009; Rosen and Harry 1990; Golub et al. 2015).

Borders of the necrotic divot caused by the impact (characterized by missing or fenestrated tissue) were estimated via comparison to the contralateral hemisphere. The surrounding tissue, marked by a high density of morphologically activated Iba1 positive cells (Goodfellow et al. 2022), was identified as the “penumbra.” IgG effusions were identified as halos of immunoreactivity surrounding microvessels; these were also quantified by the Cavalieri method. Diffuse and punctate APP immunoreactivity within white matter tracts (cortical, corpus callosum, and cingulate) chosen for proximity to the injury site was included in the total APP volume.

### 2.9 Data Analysis

Whenever possible, experimenters were blinded to treatment during data analysis. Analysis of differences between groups was conducted using one‐way and repeated measures analyses of variance (ANOVA). *Post hoc* comparisons used the Tukey's method, and differences between groups at *p* < 0.05 were considered significant. Assumptions of normality and equal variance were tested using the Shapiro–Wilks and Brown‐Forsythe methods, respectively, prior to analyzing data. In the event of a failed normality test, the Kruskal–Wallis One Way ANOVA on Ranks with Dunn's post hoc test was applied. A one‐sample *t* test was used to analyze preference in the object location/recognition tests; a treatment group PI >50 denotes a significant preference. One‐sample *t* test (or, if nonparametric, one‐sample signed rank test) was also used to assess group differences when a group mean is equal to zero with zero variability; treatment group mean > 0 denotes a significant difference. All analyses were conducted using SigmaPlot statistical software, Version 15.0 (Systat, CA). Box plots depict minimum, 1st quartile, median, 3rd quartile, and maximum.

## 3 Results

### 3.1 Blast Measurements

The peak acceleration (*g*) was significantly different between the 1.75 g and the 2.5 g charge (*F* (1, 18) = 17.58, *p* < 0.001); the 1.75 g charge resulted in a peak acceleration of 1983 ± 227 *g*, whereas the 2.5 g charge was 2422 ± 239 *g* (mean ± SD). This corresponded with a significant difference in jerk (m/s^3^) between charge levels (H (1) = 8.56, *p* < 0.003). See Table 2.

**TABLE 2:** Peak acceleration and jerk by experiment.

Trial	Charge (g)	Peak acceleration (*g*)	Peak jerk (m/s^3^)
1	1.75	1876	19,540,000
2	1.75	1548	15,390,000
3	1.75	2380	22,588,000
4	1.75	2165	19,600,000
5	1.75	2040	18,754,000
6	1.75	1754	16,500,000
7	1.75	2000	18,666,000
8	1.75	2066	18,490,000
9	1.75	1809	16,368,000
10	1.75	2032	17,490,000
11	1.75	2152	18,519,000
12	2.5	2568	28,140,000
13	2.5	2168	22,920,000
14	2.5	2860	26,200,000
15	2.5	2136	13,850,000
16	2.5	2575	26,335,000
17	2.5	2528	27,584,000
18	2.5	2354	24,664,000
19	2.5	2428	25,465,000
20	2.5	2180	24,678,000

### 3.2 Injury Induction

Table 3 demonstrates that, at baseline, body weights did not differ significantly by assigned group, though they did approach significance (*F* (3, 29) = 2.67, *p* = 0.066). Therefore, post‐injury body weight was assessed as a percent of baseline. No between‐group differences in the percent change from baseline body weight were noted (*F* (3, 29) = 1.44, *p* = 0.251).

**TABLE 3:** Body weights at baseline and 7 days post‐injury.

Group	Baseline body weight (kg)	7DPI body weight (kg)	7DPI % change in body weight
Blast	1.0 ± 0.02	1.1 ± 0.02	7.4 ± 0.8
CCI	1.0 ± 0.03	1.1 ± 0.03	6.9 ± 1.2
BCCI	1.0 ± 0.03	1.1 ± 0.03	8.1 ± 1.4
Sham	1.1 ± 0.05	1.2 ± 0.05	10.5 ± 1.1

CCI injury at a 4 mm depth in *n* = 3 animals did not result in reliable dura rupture. Therefore, the CCI depth was increased to 6 mm; animals that received a 4 mm depth injury were excluded from further analysis. For the blast, *n* = 9 animals received blast‐alone and *n* = 2 animals received BCCI with a 1.75 g PETN explosive. Unlike rats from previous studies (Proctor et al. 2017; Tchantchou et al. 2017), we did not note any immediate effects of the blast on the health of the animal (e.g., bleeding, labored breathing, etc.). Therefore, the explosive was increased to 2.5 g (the maximum possible size for our facility) for an additional *n* = 3 blast‐alone and *n* = 6 BCCI. No differences between the 1.75 g and the 2.5 g PETN blast were noted in neuroimaging, histopathology, and behavior, so these animals were combined into a single group for analysis. All animals survived until the designated terminal endpoint (7 days post‐injury).

### 3.3 Neurobehavioral Outcomes

Neurobehavioral assay protocols were refined in the early stages of the experiment, leading to finalized methods for *n* = 6 BCCI animals and *n* = 5 Sham animals for all tests except OLT/NOR, for which data is available for *n* = 4 CCI, *n* = 8 BCCI, *n* = 7 Blast, and *n* = 5 Sham, and Ladder, for which data is available for *n* = 5 BCCI and *n* = 5 Sham animals. Data collected before methodologies were finalized is not shown.

#### 3.3.1 Behavioral Observation

**FIGURE 2:**
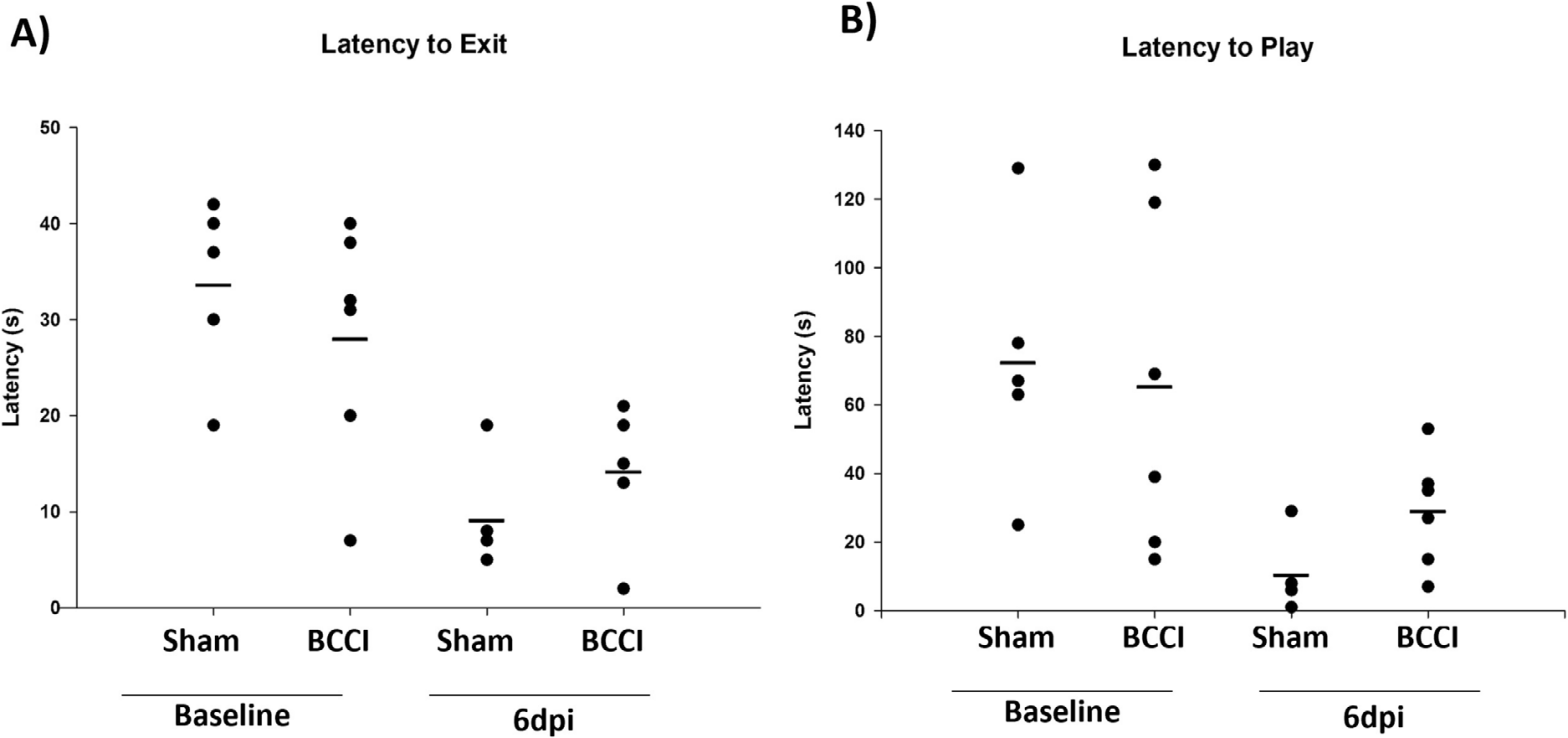
No more than 1 week pre‐injury and 6 days post‐injury, animals were released into the behavior testing suite and observed. (A) No differences were noted in latency to exit the pet carrier at baseline or post‐injury. (B) Furthermore, no differences were noted in their latency to express play behaviors at baseline or post‐injury. Horizontal lines denote group mean. *n* = 5–6 per group (one‐way analysis of variance).

A ferret solitary play behavior ethogram (see Table 1) was developed based on experimenter observation and a careful review of the literature (Goodfellow et al. 2024; Vinke and Schoemaker 2012; Fisher 2006; Bulloch and Tynes 2010; Larrat and Summa 2021; Poole 1972; Reijgwart et al. 2018). Figure 2 illustrates that no differences were detected at baseline in carrier exit (*F* (1, 9) = 0.69, *p* = 0.429) or play latency (*F* (1, 9) = 0.068, *p* = 0.800). One‐way ANOVA failed to detect differences in latency to exit the carrier at 6 days post‐injury (*F* (1, 9) = 1.63, *p* = 0.234). The latency to express at least one play behavior was marginally significantly different (*F* (1, 9) = 4.65, *p* = 0.059) with injured animals averaging a longer latency.

#### 3.3.2 Ladder Walk

**FIGURE 3:**
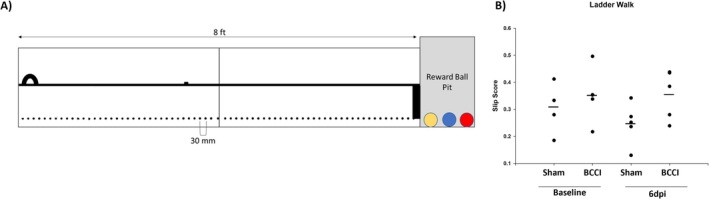
(A) No more than 1 week pre‐injury and 6 days post‐injury, animals walked across a horizontal ladder and the number and severity of slips were quantified to determine a slip score. (B) No differences were noted in slip score at baseline or post‐injury. Horizontal lines denote group mean. *n* = 5–6 per group (one‐way analysis of variance).

Figure 3 shows that, while no differences were detected at baseline (*F* (1, 8) = 0.55, *p* = 0.479), one‐way ANOVA found that the difference in slip score between groups was marginally significant (*F* (1, 8) = 4.17, *p* = 0.075) with BCCI animals, on average, performing worse than Sham animals on 6 dpi.

#### 3.3.3 Open Field

**FIGURE 4:**
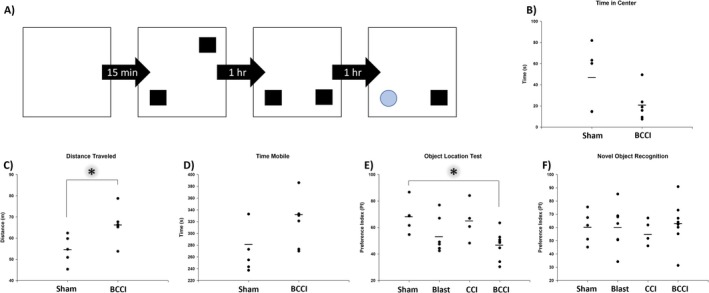
Six days post‐injury. (A) Animals underwent Open Field, followed by the Object Location Test/Novel Object Recognition (OLT/NOR). (B) While no significant differences in time spent in the center zone were noted. (C) BCCI animals traveled significantly further than Sham animals, indicative of hyperactivity. (D) No differences in time spent mobile were noted. (E) BCCI animals performed significantly worse than Sham animals on the OLT, indicative of impaired hippocampus‐dependent memory. (F) No differences were noted on the NOR. Horizontal lines denote group mean. An asterisk (*) denotes *p* < 0.05. Group sizes were Blast (*n* = 7), CCI (*n* = 4), BCCI (*n* = 8), Sham (*n* = 5) (one‐way analysis of variance with Tukey's post hoc test).

One‐way ANOVA found that BCCI animals traveled significantly further than Sham animals at 6 days post‐injury (*F* (1, 9) = 6.59, *p* = 0.030), indicating they may be hyperactive at this timepoint. Time spent mobile just missed significance (*F* (1, 9) = 4.14, *p* = 0.072) though, on average, BCCI animals spent more time in motion. This increase in activity did not correspond with increased time spent in the center zone of the arena; a marginally significant difference was noted (*F* (1, 9) = 3.41, *p* = 0.098) with Sham animals averaging more time in the center zone. See Figure 4.

#### 3.3.4 Memory Tests

Figure 4 illustrates memory test results. No differences were noted during the training phase (*F* (3, 18) = 0.76, *p* = 0.531) and, for all groups, no preference was shown for either object (one‐sample t‐tests with hypothesized PI mean = 50, all *p* > 0.05). On the Object Location Test, a significant effect of Treatment was noted (*F* (3, 20) = 3.89, *p* = 0.024). Post hoc analyses found that, relative to Sham animals, BCCI animals performed significantly worse (*p* = 0.032). On Novel Object Recognition, no significant differences were noted between groups in time spent exploring the novel object (*F* (3, 20) = 0.16, *p* = 0.922), indicating a lack of a deficit in perirhinal cortex‐dependent novelty detection in injured animals.

### 3.4 Magnetic Resonance Imaging

**FIGURE 5:**
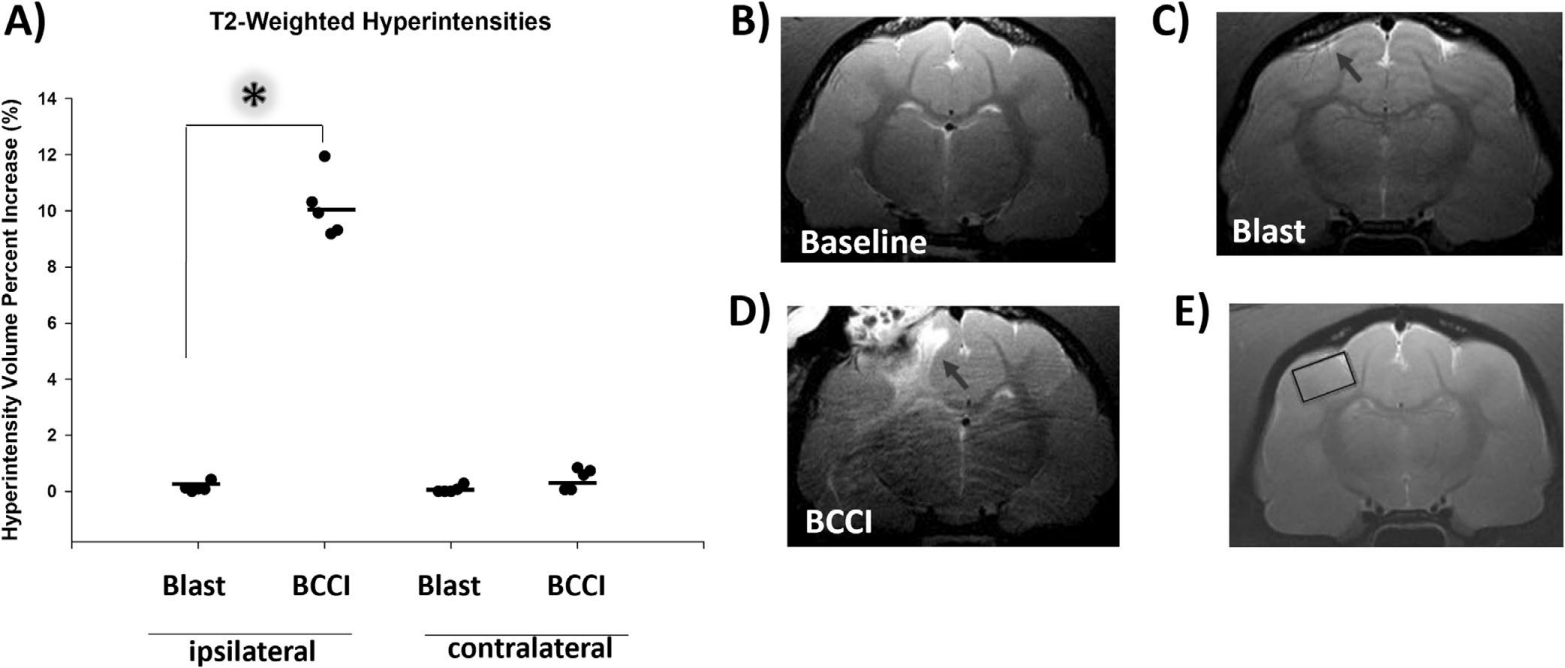
No more than 1 week pre‐injury and 7 days post‐injury, animals underwent T_2_‐weighted magnetic resonance imaging and spectroscopy. (A) Significant differences in the increase in hyperintensity volume from baseline were noted between Blast and BCCI animals ipsilateral but not contralateral to the injury. Small *x*‐axis jitter was used to avoid overplotting. Representative T_2_‐weighted MRI scans at (B) baseline, (C) in a Blast animal, and (D) in a BCCI animal. (E) The voxel location for magnetic resonance spectroscopy, indicated by a black box. An asterisk (*) denotes *p* < 0.05. Arrows indicate the location of the impact lesion or the equivalent location in Blast animals. Horizontal lines denote group mean. *n* = 5 per group (one‐way analysis of variance on ranks).

Analysis of T_2_‐weighted magnetic resonance imaging (MRI) scans 1 week post‐injury found a significant increase from baseline in the percent hyperintensity (hyperintensity volume/total cortical volume × 100) in BCCI brains ipsilateral to the injury relative to Blast (*H* (1) = 6.82, *p* = 0.008)—Figure 5. In the hemisphere contralateral to the injury, the percent increase in hyperintensity volume approached significance between groups (*F* (1, 8) = 4.83, *p* = 0.059), with BCCI animals averaging higher volumes than Blast animals. Hyperintensity is indicative of edema.

### 3.5 Magnetic Resonance Spectroscopy

**FIGURE 6:**
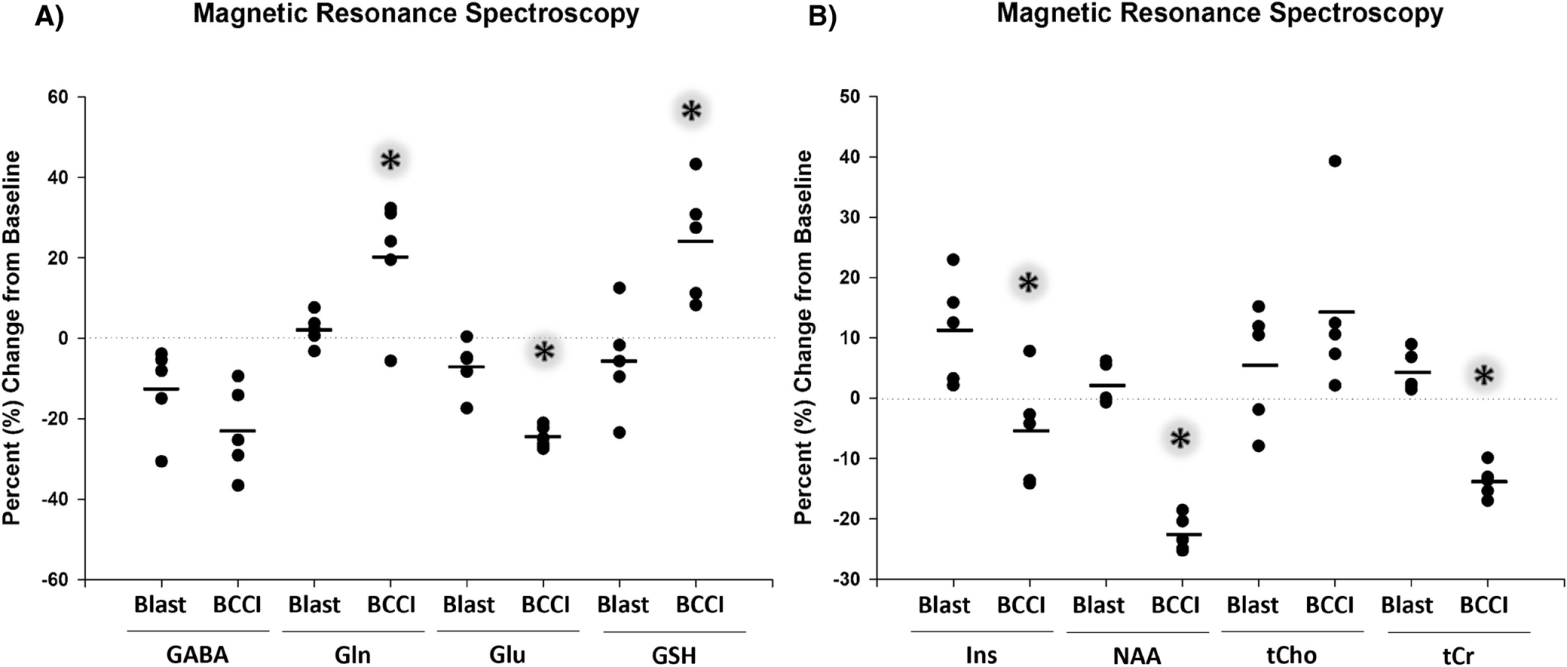
Magnetic resonance spectroscopy (MRS) percent change at 7 days post‐injury from pre‐injury baseline. (A) Depicts GABA: g‐aminobutyric acid, Gln: Glutamine, Glu: Glutamate, GSH: Glutathione. (B) Depicts Ins: *myo‐*inositol, Lac: Lactate, NAA: *N‐*acetyl aspartate, Tau: Taurine, tCho: glycerophosphocholine + phosphocholine, tCr: creatine + phosphocreatine. An asterisk (*) denotes a significant effect of Treatment × Time. Two neurochemicals (Lac and Tau) are not shown due to lack of effect. Horizontal lines denote group mean. *n* = 5/group.

A significant difference between groups was noted at baseline in glutamine (Gln; *F* (1, 8) = 6.15, *p* = 0.038), so repeated measures ANOVA was used to determine change from baseline. Table 4 shows MRS statistical analyses (repeated measures ANOVA; Treatment × Time); see Figure 5 for voxel placement and Figure 6 for results. Neurometabolic analysis showed multiple changes following a blast injury and BCCI. Neurotransmitter glutamate and GABA levels were significantly decreased from baseline at 7 days in both the BCCI and Blast conditions. Interestingly, only the BCCI group showed a concomitant increase in glutamine. Total creatine (tCr), a high‐energy phosphate buffer and alternative fuel source, shared the same post‐injury profile, with levels significantly decreasing in the BCCI and Blast groups at 7 days compared to baseline. BCCI animals also showed a significant increase in glutathione (GSH), which is linked to oxidative stress. *N*‐acetylaspartate (NAA), a marker of neuronal mitochondrial integrity, was significantly decreased in the BCCI group after injury. The BCCI group showed a marked increase in total choline (tCho), a marker of cell membrane integrity. Blast, but not BCCI, also showed a significant increase in inositol (Ins), which may indicate osmotic stress. For lactate, a marker of glycolysis, a nonparametric test on Percent Change ([1‐(7dpi Lac/baseline Lac)] × 100) was performed due to a failed normality test for the repeated measures analysis; no changes were noted, likely due to high variability within groups. Taurine, an osmoregulatory and glial cell marker, also showed no effect.

**TABLE 4:** Repeated measures analysis of variance of magnetic resonance spectroscopy in neurochemical expression, comparing Treatment (BCCI vs. Blast), Time (baseline vs. 7dpi), and Treatment × Time (except for Lac—see Section 3).

Neuro‐chemical	Comparisons	Post hoc effect narrative
GABA	Treatment *F* (1, 8) = 1.325, *p* = 0.238	GABA was significantly lower at 7dpi relative to baseline (*p* = 0.002)
	Time *F* (1, 8) = 22.497, *p* = 0.001*	
	Treatment × Time *F* (1, 8) = 1.641, *p* = 0.236	
Gln	Treatment *F* (1, 8) = 19.181, *p* = 0.002*	Gln was significantly increased in BCCI animals at 7dpi relative to baseline (*p* = 0.004). At 7dpi, Gln was significantly different in BCCI animals relative to Blast animals (*p* < 0.001)
	Time *F* (1, 8) = 9.632, *p* = 0.015*	
	Treatment × Time *F* (1, 8) = 6.45, *p* = 0.035*	
Glu	Treatment *F* (1, 8) = 11.139, *p* = 0.01*	Both Blast (*p* = 0.013) and BCCI (*p* < 0.001) animals had significant decreases in Glu on 7dpi relative to baseline. At 7dpi, Blast and BCCI expressed significantly different amounts of Glu (*p* < 0.001)
	Time *F* (1, 8) = 103.369, *p* < 0.001*	
	Treatment × Time *F* (1, 8) = 31.695, *p* < 0.001*	
GSH	Treatment *F* (1, 8) = 4.615, *p* = 0.064	At 7dpi, the amount of GSH expressed was significantly different between Blast and BCCI animals (*p* = 0.001)
	Time *F* (1, 8) = 3.929, *p* = 0.083	
	Treatment × Time *F* (1, 8) = 12.553, *p* = 0.008*	
Ins	Treatment *F* (1, 8) = 6.456, *p* = 0.035*	For Blast animals, Ins was significantly increased at 7dpi relative to baseline. At 7dpi, Blast Ins was significantly different from BCCI Ins (*p* = 0.004)
	Time *F* (1, 8) = 1.046, *p* = 0.336	
	Treatment × Time *F* (1, 8) = 9.759, *p* = 0.014*	
Lac	Failed normality test. ANOVA on ranks for Percent Change found no significant differences (*H* (1) = 1.353, *p* = 0.310)	—
NAA	Treatment *F* (1, 8) = 7.391, *p* = 0.026*	In BCCI animals, NAA was significantly decreased at 7dpi relative to baseline (*p* < 0.001). On 7dpi, NAA was significantly different between Blast and BCCI animals (*p* < 0.001)
	Time *F* (1, 8) = 89.674, *p* < 0.001*	
	Treatment × Time *F* (1, 8) = 130.858, *p* < 0.001*	
Tau	Treatment *F* (1, 8) = 1.305, *p* = 0.286	—
	Time *F* (1, 8) = 0.031, *p* = 0.865	
	Treatment × Time *F* (1, 8) = 1.58, *p* = 0.244	
tCho	Treatment *F* (1, 8) = 2.884, *p* = 0.128	tCho levels were significantly increased at 7dpi relative to baseline (*p* = 0.031)
	Time *F* (1, 8) = 6.812, *p* = 0.031*	
	Treatment × Time *F* (1, 8) = 1.409, *p* = 0.269	
tCr	Treatment *F* (1, 8) = 6.665, *p* = 0.033*	Relative to baseline, tCr was significantly different in both Blast (increased, *p* = 0.018) and BCCI (decreased, *p* < 0.001) animals. At 7dpi, Blast and BCCI animals expressed significantly different levels of tCr
	Time *F* (1, 8) = 23.433, *p* = 0.001*	
	Treatment × Time *F* (1, 8) = 81.8, *p* < 0.001*	

*Note:* In the Comparisons column, the repeated measures ANOVA statistics are given. Significant effects are noted with an asterisk (*p < 0.05) and highlighted in gray. The Post Hoc Effect Narrative column describes the outcomes of Tukey's post hoc tests, which were performed in the case of a significant main effect or interaction. *n* = 5 per group.

Abbreviations: GABA, G‐aminobutyric acid; Gln, Glutamine; Glu, Glutamate; GSH, Glutathione; Ins, *Myo‐*inositol; Lac, Lactate; NAA, *N‐*acetyl aspartate; Tau, Taurine; tCho, Glycerophosphocholine + phosphocholine; tCr, Creatine + phosphocreatine.

### 3.6 Histopathology

#### 3.6.1 Iba1

Stereological analysis of divot volume (missing + fenestrated tissue) found a significant effect of Treatment (*F* (2, 15) = 7.91, *p* = 0.005) with CCI (*p* = 0.018) and BCCI (*p* = 0.005) significantly different from Sham; BCCI and CCI did not differ significantly from each other (*p* = 0.945). Penumbra was also found to have a significant effect of Treatment (*F* (2, 15) = 16.85, *p* < 0.001). Post hoc tests revealed a significant difference between CCI versus Sham (*p* < 0.001) and BCCI versus Sham (*p* < 0.001), but not CCI versus BCCI (*p* = 0.535).

**FIGURE 7:**
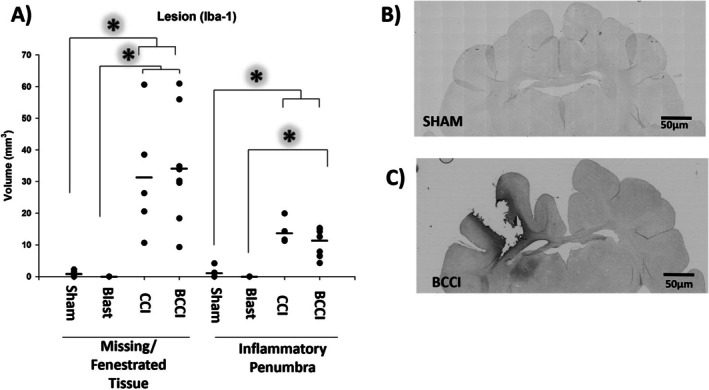
Iba1 immunohistochemistry at 7 days post‐injury. Relative to Sham animals, CCI and BCCI animals show significantly greater volumes of (A) missing/necrotic tissue (divot; one‐way analysis of variance with Tukey's post hoc) and dense Iba1+ immunoreactivity around the divot (inflammatory penumbra; one‐way analysis of variance with Tukey's post hoc test). Blast animals were found to be significantly different from CCI and BCCI groups in divot volume but only BCCI animals for penumbra volume (one‐sample *t*‐test). Representative photomicrographs (5×) from (B) a Sham and (C) a BCCI animal. An asterisk denotes statistical significance (*p < 0.05). Horizontal lines denote group mean. Group sizes were Blast (*n* = 10), CCI (*n* = 5), BCCI (*n* = 8), and Sham (*n* = 5).

For comparisons with Blast animals for divot and penumbra, which had a group mean volume of zero, a one‐sample t‐test or, in the case of failed normality test, a one sample signed rank test, was used to determine if the other treatment group means differed from zero. BCCI animals were found to have volumes significantly greater than zero for both divot (*t* (7) = 5.58, *p* < 0.001) and penumbra (*t* (7) = 5.58, *p* < 0.001). In CCI animals, the divot was significantly greater than zero (*t* (4) = 3.643, *p* < 0.05) but the penumbra was not (*Z* = 2.02, *p* = 0.063). Sham animals were found to have mean volumes that did not differ significantly from zero for both divot (*t* (4) = 1.92, *p* = 0.064) and penumbra (*Z* = 1.34, *p* = 0.500). Results indicate that, for the divot, BCCI and CCI animals were significantly different from Blast. For penumbra, only BCCI animals were significantly greater than Blast. See Figure 7.

#### 3.6.2 IgG

**FIGURE 8:**
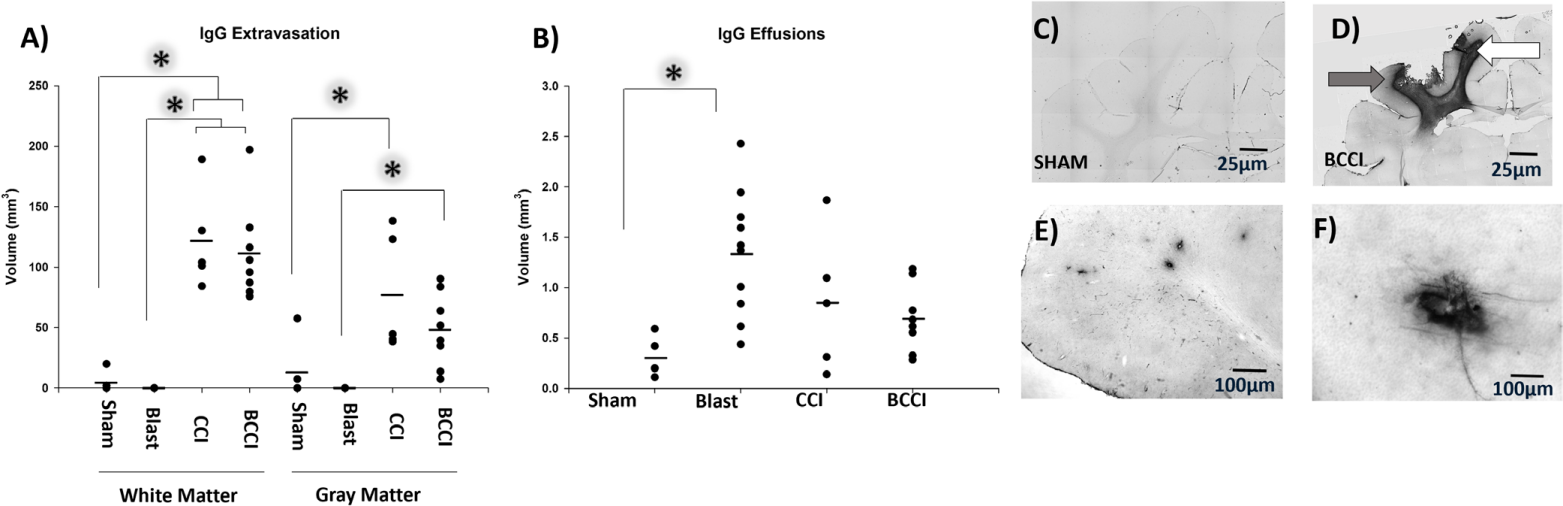
Immunoglobulin G (IgG) immunohistochemistry at 7 days post‐injury. Volume of IgG+ tissue was significantly increased in CCI and BCCI animals relative to Blast and Sham animals in (A) white matter. In gray matter, CCI was significantly greater than Sham whereas BCCI was significantly greater than Blast. (one‐way ANOVA with Tukey's post hoc, one‐way analysis of variance on ranks with Dunn's post hoc, one‐sample *t*‐test). (B) IgG effusions from blood vessels were significantly increased in blast animals relative to BCCI and Sham animals (one‐way analysis of variance with Tukey's post hoc test). Representative photomicrographs (5×) from (C) a Sham and (D) a BCCI animal; the white arrow indicates the white matter, and the gray arrow indicates the gray matter. Examples of IgG effusions are shown at (E) 5× and (F) 40×. An asterisk denotes statistical significance (*p < 0.05). Horizontal lines denote group mean. Group sizes were Blast (*n* = 10), CCI (*n* = 5), BCCI (*n* = 8), and Sham (*n* = 5).

A significant effect of Treatment was found in IgG effusions (*F* (3, 24) = 5.12, *p* = 0.007) where exposure to Blast increases IgG effusions relative to Sham animals (*p* = 0.006); the difference between Blast and BCCI approached significance (*p* = 0.064). A significant effect of Treatment in white matter IgG was noted (*H* (2) = 10.41, *p* = 0.005) with both BCCI and CCI significantly different from Sham (*p* < 0.05). In terms of gray matter, a significant effect of Treatment was also noted (*F* (2, 15) = 4.12, *p* = 0.038) with CCI animals significantly different from Sham (*p* < 0.05). For comparisons with Blast animals in white and gray matter IgG, which had a group mean volume of zero, a one‐sample *t*‐test or, in the case of a failed normality test, a one‐sample signed rank test, was used to determine if the other treatment group means differed from zero. BCCI were found to be significantly greater than zero for both white (*t* (7) = 7.96, *p* < 0.001) and gray matter (*t* (7) = 4.50, *p* < 0.01). For CCI, only white matter was significantly different from zero (*t* (4) = 6.61, *p* < 0.01); gray matter failed to achieve significance (*Z* = 2.02, *p* = 0.063). See Figure 8.

#### 3.6.3 Amyloid Precursor Protein

**FIGURE 9:**
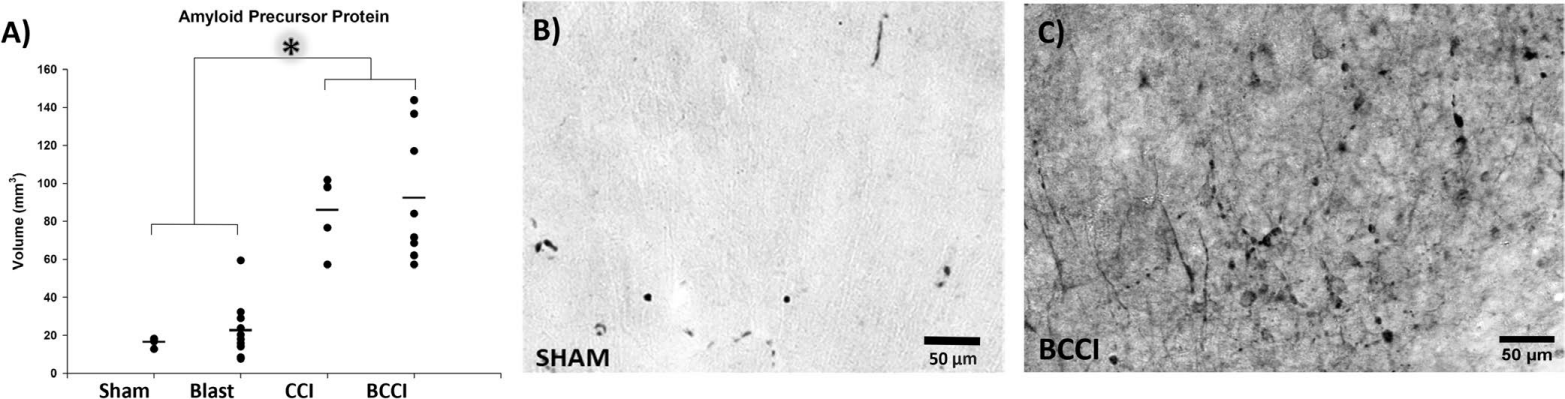
Amyloid precursor protein (APP) immunohistochemistry at 7 days post‐injury. (A) Relative to Blast and Sham, CCI and BCCI animals had significantly greater APP+ volume. Representative photomicrographs (20×) from (B) a Sham and (C) a BCCI animal. An asterisk denotes statistical significance (*p < 0.05). Horizontal lines denote group mean. Group sizes were Blast (*n* = 10), CCI (*n* = 5), BCCI (*n* = 8), and Sham (*n* = 5) (one‐way analysis of variance with Tukey's post hoc test).

Figure 9 illustrates punctate and diffuse APP positive staining in transverse and longitudinal fibers throughout the gray and white matter of the cortex near the site of impact. Stereologic analysis of APP revealed that exposure to a single under vehicle blast did not worsen diffuse axonal injury measured 7 days later compared to Sham groups (*F* (3, 24)= 22.59, *p* < 0.001). CCI and BCCI groups had significantly more APP+ staining than Blast or Sham (*p* < 0.001).

## 4 Discussion

In this pilot study, we sought to develop a military‐relevant model of under‐vehicle blast TBI in ferrets as well as methodologies to measure injury outcomes (MRI/MRS, behavior, histopathology). We compared neurologic outcomes between blast‐alone, CCI‐alone, and combined blast + CCI models. Results demonstrate differential structural and functional outcomes in these models.

### 4.1 Under‐Vehicle Blast‐Induced Neuropathology

Despite a trend towards impairment in spatial memory on the Object Location Test, under‐vehicle blast animals showed very subtle neuropathological changes. MRS demonstrated alterations relative to baseline in several neurochemicals, including markers of altered neurotransmission, energy metabolism, and osmotic stress. Histologically, only IgG effusions from blood vessels were significantly increased in blast‐alone animals relative to Sham animals. In a ferret model of blast overpressure exposure, injured animals show changes in astroglial morphology and MRI hypointensities around blood vessels at 12 weeks post‐injury, suggestive of vascular injury (Schwerin et al. 2021). In line with our MRS findings at 7 days post‐injury with under‐vehicle blast, blast overpressure induced MRS‐detectable changes in glutamate. However, it did not cause similar changes in GABA, total creatine, and inositol (Tang et al. 2024). This may be due to differences in injury or voxel placement.

Previous work from our lab (Tchantchou et al. 2017) investigating under‐vehicle blast injury in rodents found spatial working memory (Y‐maze) impairments 1 week post‐injury. This aligns well with the results of the present study where we detected marginally significant effects of blast on spatial memory, despite being statistically underpowered. In both the rats (Tchantchou et al. 2017) and the ferrets, blast induced vascular injury. However, while significant evidence of increased inflammatory processes was evident in rats 1 day post‐injury, this was not observed at 7 days post‐injury; the ferrets also failed to show signs of inflammation 1 week after injury. In terms of mortality, both rats and ferrets survived blasts resulting in about 2400 G. In rats, mortality (67%) occurred only when the mean maximal force generated was about 2800 G, suggesting mortality in ferrets may also occur at higher G force. Taken together, these findings indicate that under‐vehicle blast induces acute/subacute neuropathology in both rats and ferrets.

### 4.2 CCI‐Induced Neuropathology

Limited results are available for CCI‐alone animals. CCI‐alone did not result in impaired spatial memory or novelty detection, which aligns with previous findings in ferrets 1 week after CCI (Schwerin et al. 2018). Furthermore, relative to Sham animals, CCI did not induce a significantly larger divot, nor did it increase IgG immunoreactivity in white and gray matter. This lack of statistical significance is likely due to the small n and large variability for the CCI group. Despite this, CCI did result in significant diffuse axonal injury and microglial activation.

Previous work in ferrets has shown a significant increase in Iba1 relative to naïve controls at 1 week after CCI (Schwerin et al. 2018), which is consistent with our findings. Furthermore, we previously demonstrated that craniotomy alone induces a mild TBI, evidenced by the presence of increased Iba1 immunoreactivity near the craniotomy site (Goodfellow et al. 2022). Indeed, unlike craniotomized animals, those in the under‐vehicle blast alone group, who did not receive a craniotomy, show a lack of Iba1 inflammatory lesion and very little IgG immunoreactivity in cortical tissue.

### 4.3 Combined Under‐Vehicle Blast and CCI Neuropathology

Relative to blast, the combination of under‐vehicle blast and CCI led to additional neurochemical changes on MRS relative to baseline, including an increase in markers of oxidative stress and cell membrane integrity and a decrease in a marker of neuronal integrity. This aligns with findings in this model at a similar timepoint using metabolic imaging of hyperpolarized [1‐13C]pyruvate (Mayer et al. 2025). Despite relatively small group sizes, we demonstrate neurobehavioral impairments in BCCI animals relative to Sham controls that achieve or approach statistical significance, including mood, motor function, and spatial memory; larger group sizes are necessary for future studies for adequate statistical power. These functional deficits are accompanied by increased edema, as well as a large necrotic divot and inflammatory penumbra, increased blood–brain barrier permeability in both white and gray matter, and axonal injury. Results contrast with research in CCI‐alone ferrets who did not demonstrate significant alterations in behavior assays (motor function, novelty detection, anxiety‐like behavior), except for working memory, at 1 week post‐injury (Schwerin et al. 2018). This may be due to the addition of the blast injury and/or methodological differences in behavioral assessment.

Schwerin et al. (2023) used a related model in which ferrets underwent shockwave blast plus closed‐head impact injury. In this model, they noted altered activity patterns relative to Sham animals at 4 weeks post‐injury. However, unlike our observations of increased activity in the open field, they showed high activity levels decrease after injury. This could be due to differences in the injury model, type of observation (extended actiography vs. a single, discrete observation), or the timing of the observation (1 week vs. 4 weeks post‐injury). Histologically, injured animals showed increased astrogliosis (GFAP) and aquaporin 4 expression at 4 weeks post‐injury, which corresponds with our findings at 1 week post‐injury of ongoing injury/repair processes.

## 5 Limitations

Given its nature as a pilot study, there are several limitations of note. Assays were largely underpowered and, in some cases, data trends did not provide statistically significant results. Larger sample sizes would clarify any effects. The behavioral and neuroimaging results are limited to a subset of treatment groups, making it difficult to tease apart the effects of blast and CCI alone relative to the combined injury. The use of a single subacute timepoint provides very limited information. Finally, while the similarities between the human and ferret brain provide this model with excellent face validity, most human studies of blast injuries investigate chronic outcomes. Therefore, studies that comprehensively assess long‐term neurobehavioral, imaging, and histopathologic outcomes after blast exposure are needed to fully validate the ferret model (Saar‐Ashkenazy et al. 2023; Coppel et al. 2024; Lange et al. 2024).

The study was also only performed in young males; given the many sex differences that have been identified after TBI (Bazarian et al. 2010; Späni et al. 2018), results may not be translatable to both sexes. Unfortunately, the ferret estrous cycle poses unique challenges for researchers (Jekl and Hauptman 2017); investigations into potential cycle management strategies are necessary to advance the ferret model for use in both sexes. Age could also influence pathophysiology; the ferrets in this study were quite young at the time of injury (~10 weeks of age). Future studies should investigate age as a variable.

## 6 Conclusions and Future Directions

The results of this study demonstrate that under‐vehicle blast‐alone, CCI‐alone, and blast + CCI result in unique neuropathology in the gyrencephalic brain. Ferrets can provide rich neurobehavioral, imaging, and histopathologic outcomes for brain injury research. The methodology developed in this study will be applied to future military‐ and civilian‐relevant investigations into the impact of TBI on the gyrencephalic brain.

## 7 Transparency, Reproducibility, and Rigor

The study design and analytic plan were not pre‐registered. As this was a pilot study, power analyses were not performed to determine sample size. A total of 33 ferrets were used, and ferrets were assigned to groups based on when they were received in the experimental timeline (group sizes as stated in Section 2). Behavior data collected before behavior assay protocols were finalized is excluded. No data was excluded from neuroimaging analyses. A subset of CCI animals (*n* = 3) were excluded because of the lack of dura rupture. Group sizes varied by outcome measure: behavior (*n* = 4–8‐group), neuroimaging (*n* = 5/group), and histology (*n* = 5–10/group). All antibodies and reagents used in this study are commercially available. Investigators were blinded to treatment groups to the fullest extent possible for the analysis of animal behavior, neuroimaging, and histology. Open Field data was automatically quantified by AnyMaze. Assumptions of normality and equal variance were tested using the Shapiro–Wilks and Brown‐Forsythe methods. When data sets failed these tests, nonparametric analyses were applied. The data sets are available from the corresponding author upon reasonable request and with approval from the United States Air Force.

## Author Contributions

Conceptualization: J.L.P., R.P.G., U.H.L., W.L.F., C.H.T.M., G.F. Methodology: M.J.G., A.L.H., B.P., J.L.P., P.R., S.X., U.H.L., W.L.F. Validation: M.J.G., J.L.P., P.R., S.X., U.H.L., W.L.F. Formal analysis: M.J.G., B.P., J.L.P., P.R., S.X. Investigation: M.J.G., A.L.H., B.P., J.L.P., P.R., M.C.S., A.V., S.X., U.H.L., W.L.F. Writing – original draft: M.J.G., A.L.H., S.X. Writing – review and editing: M.J.G., J.L.P., U.H.L., W.L.F., G.F. Visualization: M.J.G., J.L.P. Supervision: M.J.G., J.L.P., G.F. Project administration: J.L.P., C.H.T.M., J.C.C., and funding acquisition: C.H.T.M., G.F.

## Disclosure

The views expressed are those of the authors and do not reflect the official guidance or position of the United States Government, the Department of Defense, or of the United States Air Force. Imagery in this document is property of the U.S. Air Force. AFRL‐2025‐0459, cleared 2 June 2025.

## Conflicts of Interest

The authors declare no conflicts of interest.

## Declaration of Transparency

The authors, reviewers and editors affirm that in accordance to the policies set by the *Journal of Neuroscience Research*, this manuscript presents an accurate and transparent account of the study being reported and that all critical details describing the methods and results are present.

## Supporting information


**Data S1:** jnr70081‐sup‐0001‐DataS1.docx.

## Data Availability

Data from this study are available upon reasonable request and with approval from the United States Air Force.
